# P46 A rare case of costovertebral arthritis in SLE

**DOI:** 10.1093/rap/rkac067.046

**Published:** 2022-09-28

**Authors:** Afzal Latheef, Jomine James Puthupparmbil, Nidhi Sofat

**Affiliations:** St George's University Hospitals NHS Foundation Trust, London, United Kingdom; St George's University Hospitals NHS Foundation Trust, London, United Kingdom; St George's University Hospitals NHS Foundation Trust, London, United Kingdom

## Abstract

**Introduction/Background:**

Systemic Lupus Erythematosus (SLE) is a chronic, multi-system, autoimmune condition with a waxing and waning course affecting joints, skin, hair, heart, lungs and kidneys.The atypical activation of native immunity in SLE causing immune complexes deposition throughout the body directly induces inflammation and tissue damage.

SLE arthritis usually follows non-erosive pattern. The costovertebral joints are affected when there is mechanical irritation between the spinal curve at T6-T8 and oblique and twisting ribs, causing localised and referred pain to chest and abdomen when intercostal nerves are involved.

We present an unusual case of chronic back pain from thoracic costovertebral arthritis secondary to SLE.

**Description/Method:**

This 32-year-old black woman presented to rheumatology seven years ago, with a few years’ history of alopecia, mouth ulcers, mid-thoracic back pain, intermittent chest pain, migrating polyarthralgia and fatigue on a background of Raynaud’s phenomenon, migraine, seborrheic dermatitis, and depression.

Her back pain started ten to fifteen years ago. The pain was focused on the posterior left mid thoracic region with varying intensity. It persisted throughout the day and often radiated to her anterior chest wall. The pain was aggravated by climbing stairs and other activities requiring respiratory effort.

Her initial autoimmune blood results showed a strongly positive ANA and positive Anti-Ro levels, favouring the diagnosis of SLE and Sjogren’s syndrome. She had a negative dsDNA antibody, crythidia antibody, rheumatoid factor, and normal complement levels.

She was started on Hydroxychloroquine and Non-steroidal Anti-inflammatory drugs (NSAIDS). Later on, Mycophenolate Mofetil was added following disease flare.

In the last seven years, she presented with back pain and chest pain multiple times to her primary health physician and emergency, where the diagnosis was put down to mechanical back pain and costochondritis. She was also extensively investigated by cardiology previously, for serositis with an echocardiogram, 24-hour Holter tape and cardiac MRI, all of which were normal. Later, it was thought that her symptoms were secondary to fibromyalgia and was referred to a pain specialist and for local steroid injection, which yielded no benefit.

A further rheumatology review helped to re-evaluate the clinical condition, indicating tenderness over left T4 and T5 paraspinal region along with left anterior rib margin tenderness. Further CT and MRI imaging, specialist musculoskeletal radiologist review and multidisciplinary team input revealed costovertebral arthritis secondary to lupus causing back pain and referred pain to chest wall. She has been referred for a focused steroid injection along with physiotherapy.

**Discussion/Results:**

The prevalence of chronic back pain is around 40% in the United Kingdom. Thoracic involvement is more frequently seen with SLE than any other autoimmune disease. This interesting case of chronic back pain with referred pain has a wide array of differentials and a rare finding, especially with a diagnosis of SLE. Normal chest x-ray, cardiac investigations, inflammatory markers, chronicity, and lack of infective symptoms ruled out metabolic, cardiac, and infective causes

Costovertebral and costotransverse arthritis are often associated with ankylosing spondylitis and osteoarthritis but very rarely with lupus and is an uncommon cause of thoracic backache. Fibrositis causing back pain and referred pain has more association with lupus and is seen in around 20% of SLE patients.

CT scan showed a focal soft tissue swelling in the left T8 and T9 posterior intercostal space involving the adjacent costovertebral and costotransverse joints, associated with left T8 costotransverse joint marrow signal abnormality and joint oedema. Her T1 weighted MRI and Short Tau Inversion Recovery (STIR) imaging confirmed the fat density, diffuse oedema seen with in the soft tissue mass. Her MRI was compared to previous imaging done a few years ago and interestingly showed focal swelling, oedema, and marrow signal change.

The fat swelling and oedema progressed little over time, but the marrow signal change appeared less intense on the recent MRI. The MRI also demonstrated the undistorted, uninvaded course of nerves and bloods vessels clean through the focal structure ruling out a tumour. Absence of bony spur and enthesitis ruled out ankylosing spondylitis. The absence of neurological symptoms and radiological evidence also ruled out myelitis. The detection of bone marrow oedema by MRI can be considered as a valuable tool for defining the severity and extent of inflammatory processes in chronic joint disease.

**Key learning points/Conclusion:**

This rare case cites the importance of exploring pathophysiology of chronic back pain especially in a multisystem disease like SLE. The musculoskeletal structures of the thoracic wall and the neck are a relatively common source of back pain as well as chest pain. This case helped to reinforce the fundamentals of clinical practice, careful analysis of the history, physical findings and radiological investigations are essential for precise diagnosis and effective treatment.

Also, the knowledge of the anatomic structures and thorough evaluation of the ribs, intercostal spaces, muscles, sternum, sternoclavicular joints, and cervical and thoracic spine is essential for understanding the pathophysiology of referred pain. The full facet joints T1(first thoracic vertebrae), T11 and T12 joints have high frequency for arthritis due to the absence of intervertebral discs. The peak of the spinal curve at T6-T8 corresponds to the level of oblique and twisting ribs causing more mechanical irritation between the vertebra and the ribs, leading to changes in the costovertebral joints. From a clinical perspective, patients would experience pain and local tenderness near the vertebral column aggravated by coughing and respiratory movements. The close relationship with intercostal nerves can trigger referred pain to the chest or abdomen.

This case also highlights the importance of CT and MRI imaging to establish the cause and offer effective treatment. Refractory cases of costovertebral arthritis often need local steroid injection for symptom management, biologics and rarely need resection arthroplasty. Nonetheless, the MDT approach is often valuable in favouring a conclusive diagnosis and focused treatment plan.

From the literature search we have performed, this is one of the few such reported cases. Failure to appreciate such a diagnosis could lead to continuity of symptoms, deterioration in mobility and quality of life.

